# Let’s Dance Together: Synchrony, Shared Intentionality and Cooperation

**DOI:** 10.1371/journal.pone.0071182

**Published:** 2013-08-07

**Authors:** Paul Reddish, Ronald Fischer, Joseph Bulbulia

**Affiliations:** 1 School of Psychology, Victoria University of Wellington, Wellington, New Zealand; 2 Laboratory for Experimental Research of Religion, Masaryk University, Brno, Czech Republic; 3 School of Art History, Classics and Religious Studies, Victoria University of Wellington, Wellington, New Zealand; Hungarian Academy of Sciences, Hungary

## Abstract

Previous research has shown that the matching of rhythmic behaviour between individuals (synchrony) increases cooperation. Such synchrony is most noticeable in music, dance and collective rituals. As well as the matching of behaviour, such collective performances typically involve shared intentionality: performers actively collaborate to produce joint actions. Over three experiments we examined the importance of shared intentionality in promoting cooperation from group synchrony. Experiment 1 compared a condition in which group synchrony was produced through shared intentionality to conditions in which synchrony or asynchrony were created as a by-product of hearing the same or different rhythmic beats. We found that synchrony combined with shared intentionality produced the greatest level of cooperation. To examinef the importance of synchrony when shared intentionality is present, Experiment 2 compared a condition in which participants deliberately worked together to produce synchrony with a condition in which participants deliberately worked together to produce asynchrony. We found that synchrony combined with shared intentionality produced the greatest level of cooperation. Experiment 3 manipulated both the presence of synchrony and shared intentionality and found significantly greater cooperation with synchrony and shared intentionality combined. Path analysis supported a *reinforcement of cooperation model* according to which perceiving synchrony when there is a shared goal to produce synchrony provides immediate feedback for successful cooperation so reinforcing the group’s cooperative tendencies. The reinforcement of cooperation model helps to explain the evolutionary conservation of traditional music and dance performances, and furthermore suggests that the collectivist values of such cultures may be an essential part of the mechanisms by which synchrony galvanises cooperative behaviours.

## Introduction

In all cultures around the world [1]–[3] and far back into human history [4], [5], people have come together to sing and dance. Why do humans perform such behaviour? One popular explanation is that collective music and dance bonds people together and increases cooperation [6]–[15]. Such an important function in human sociality could explain music and dance’s ubiquity and its selection and retention. Observations from ethnographies [16]–[21], historical analysis [22], [23], and experimental research [24], [25] support this social bonding hypothesis. However, it is not yet clear through what mechanism music and dance might produce this effect.

Despite variance in expressions and contexts of collective music and dance performances, one common underlying factor found in most forms of collective music and dance is the matching of rhythmic behaviours amongst performers. Performers move their bodies or produce sound at the same frequency (*frequency-locked synchrony*) or phase (*phase-locked synchrony*) [26]. Behavioural synchrony has been linked with greater social bonding and cooperation suggesting that such synchrony could be one of the key mechanism behind collective music and dance’s prosocial effect [27]–[36].

Previous research on synchrony has predominantly been conducted on pairs (dyads), (cf. [37], [38]) modelling the spontaneous and automatic synchrony that often occurs when two people interact [39]–[45]. However, the synchrony that occurs in music and dance is not accidental, but rather a deliberate communal practice. In collective music and dance, performers intentionally modify the timing of their rhythmic movement to entrain to the behaviour of others with the knowledge that others also share the goal of entrainment. This sharing of psychological states enabling collaborative behaviours is termed *shared intentionality* [46], [47]. Kirschner and Tomasello proposed that it is shared intentionality that is essential in creating the cooperative effects of collective music and dance, "getting people to experience each other as co-active, similar and cooperative members of a group” p 362 [24].

To further understanding of how collective music and dance influences cooperation, we conducted three studies designed to disentangle the social effects of synchrony and shared intentionality. In Experiment 1, we compared a condition in which shared intentionality was used to create synchrony with conditions in which synchrony and asynchrony were created as a by-product of hearing the same or different rhythmic beats. Our results from Experiment 1 helped to clarify whether shared intentionality is an important factor in producing cooperative effects from group synchrony. In Experiment 2, we measured cooperation after varying intentional synchrony with intentional asynchrony. This helped us to understand whether synchrony matters to cooperation. In Experiment 3 we manipulated both shared intentionality and synchrony to examine how their interaction affected cooperation.

## Experiment 1

For Experiment 1, we replicated and extended a recent study from Wiltermuth and Heath [37]. We manipulated synchrony by asking participants to move in time with the same rhythmic stimulus (synchrony condition), rhythmic stimuli of different frequencies (asynchrony condition), or passive non-movement (passive condition). The crucial difference in our experiment was that we included a condition that used shared intentionality to create synchrony (shared goal condition). We created shared intentionality by providing participants with a shared goal: participants were asked to work together to move in time with each other. We then measured cooperative behaviour using a public goods game. Similar to Wiltermuth and Heath, we also measured self-reported prosociality: perceived similarity, interdependent self-construal, trust, and entitativity (the degree to which a collection of people are perceived as a group [48]).

Considering previous research incorporating synchrony and shared intentionality [24], ethnographic and historical accounts of naturally occurring synchronous activities [14], [17], [18], [22], [23], and research showing that collective goals increase cooperation [49], we hypothesised that synchrony created through shared intentionality (the shared goal condition) would produce the highest level of cooperation (both behavioural and self-reported). Based on Wiltermuth and Health’s [37] findings, along with other research on incidentally induced synchronous movement [30], [34], we expected that the synchrony condition would show higher levels of cooperation compared to passive non-movement and incidentally induced asynchronous movements, but lower levels of cooperation than the shared goal condition. We therefore also predicted a linear increase of cooperation from the control and asynchrony conditions producing the lowest cooperation, to the synchrony condition, to the shared goal condition.

## Methods

### Participants

Participants were 123 volunteers recruited through posters placed around the Victoria University of Wellington New Zealand campus (85 female; mean age = 23.13 years, range: 17–42 years). All participants were paid in cash at the end of the experiment with all participants receiving a $5 show up fee (the currency for all experiments was the New Zealand dollar) and the accurate amount they earned from the economic game.

### Ethics Statement

For all three experiments reported, ethical approval was obtained from the School of Psychology Human Ethics Committee at Victoria University of Wellington. The School of Psychology Human Ethics Committee granted us the use of participants recruited from the Victoria University of Wellington student population and did not require parental or guardian consent for those below 18. All participants provided written informed consent.

### Procedure

#### Synchrony manipulation

A between subjects design was used with four conditions of the movement manipulation (shared goal, synchrony, asynchrony, and passive). For the synchrony and asynchrony conditions, participants in groups of four were asked to perform three different simple movements each lasting two minutes: (1) move their left arm up and down, then their right arm up and down; (2) sway their upper body from an upright position to the left and up, then to the right and up; and (3) move their left leg up from a standing position and down, then their right leg up and down (movements adapted from [50]). The activity took a total of six minutes. Participants were asked to move in time with a metronome beat played through headphones. We used a simple metronome beat rather than a national anthem (as used by Wiltermuth and Health [37]) to better isolate the effect of synchrony. Participants were told that the goal of the task was “to keep in time with the rhythmic pulse playing through your headphones”. In the synchrony condition, a four-way headphone splitter allowed group members to hear the same metronome beat, which was played through their headphones (tempo at 65 beats per minute [bpm]). In the asynchrony condition, a slightly different tempo (60 bpm, 65 bpm, 70 bpm, and 75 bpm) was channelled to each participant’s headphones.

The procedure of the synchrony plus the shared goal condition was the same as the synchrony condition except participants were not given a metronome beat by which to coordinate their actions. Instead, participants were told that the goal of the task was to “work together to keep in time with each other”. Participants were given a brief 10 second practice to check that everyone was able to perform movements in time. They were told when to start and when to stop, and after each two minute interval they were instructed when to change movements (arm swinging, body swaying, and stepping).

In the passive condition, a video was created of four people doing the same movement activity in synchrony with an audible metronome click in the background. Participants sat in a row on four chairs whilst watching this video for approximately six minutes. We used a video to be able to assess the impact of performing synchrony versus observing synchrony, as observation of synchrony has been found to prime entitativity [51]–[53].

#### Cooperation measure

We used a public goods economic game to test cooperation behaviourally. Participation in the game took place directly after the synchrony activity. The four participants were seated in chairs facing separate walls to minimise any communication. Participants were told they would be given $5.00 ($NZ 5 was at the time of the experiment about $US 4, roughly enough for a student lunch) for which they could contribute some or all of this $5 to a group investment. All money in the group investment would then be doubled and divided equally among all members of the group. Instructions were read out by the experimenter and were also written on a sheet handed to participants. Thus, the instructions were known to be common knowledge [54]. Participants were then handed a slip in a lettered envelope on which they confidentially indicated how much, if any, of their money they would like to contribute to the group investment (in $0.50 increments). Envelopes were then collected and taken to an assistant in another room to calculate each player’s earnings. Earnings were placed back in the same lettered envelope for participants to collect at the end of the experimental session, ensuring that participants’ contributions remained confidential. Participants received the correct amount of money they earned (rounded to the nearest $0.10).

#### Post-activity questionnaire

After the cooperation measure, participants completed a questionnaire measuring self-reported prosocial measures and demographics. Entitativity, perceived similarity and trust were measured with the same single item 7-point Likert scales as used by Wiltermuth and Heath [37]. The questions were given as follows: “How much did you feel you were on the same team with the other participants?”; “How similar do you think you are to the other participants?”; “How much did you trust the other participants going into the group investment exercise?” Interdependent self-construal was measured using an adapted version of the Inclusion of the Other in the Self scale (IOS [55]). We asked participants to rate “how close you currently feel to all the people you just did the activities with” by indicating which of a series of increasingly inclusive circles best described their judgment. Two control variables were also measured: perceived difficulty of the movement activity, which was measured used the single item “the first group activity was difficult to do” from 1 (*strongly disagree*) to 7 (*strongly agree*); and how well participants knew each other before the study, which was measured on a 5-point Likert scale from 1 (*I had never seen him/her before*) to 5 (*I know him/her very well*).

## Results

One group was removed from further analyses because three out of the four group members indicated that they were very close friends. For the remainder of the 119 participants, there were no significant differences across conditions in how well participants knew each other before the study (*F*_max_ = .89, *p* = .45).

Across all conditions, the amount of money placed in the investment ranged from $0 to $5. Results were however strongly positively skewed in the shared goal and synchrony conditions, where 55% in the shared goal condition and 50% in the synchrony condition donated the full $5 to the group investment. By contrast, 23% of participants in the asynchrony condition and 33% of participants in the passive condition donated the full $5. Shapiro-Wilk normality tests revealed that the data was strongly non-normal for both the shared goal condition, *W*(29) = .74, *p*<.001, and the synchrony condition, *W*(30) = .80, *p*<.001. The skew was also highly significant in the shared goal condition (skew = −1.31, *SE* = .43, *z* = −3.05, *p*<.01), therefore, we used non-parametric statistics to test the effect of our manipulations. A Kruskal-Wallis test found a marginally significant difference in mean ranks across conditions, *H*(3) = 6.94, *p* = .07.

**Figure 1:**
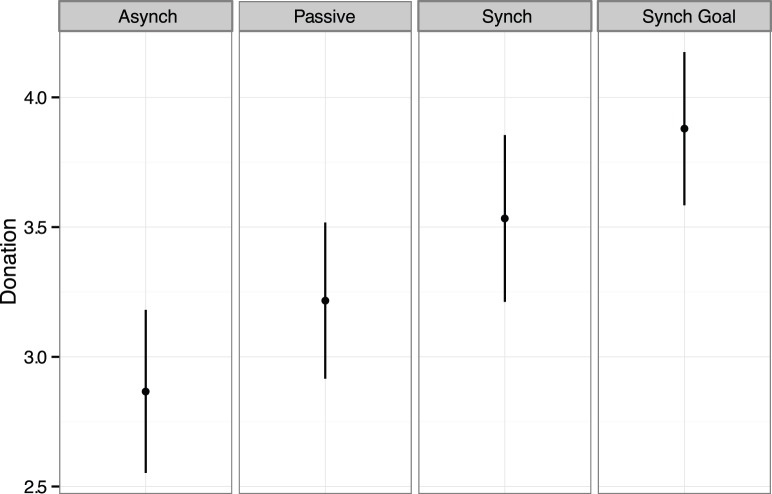
Cooperation across experimental conditions for Experiment 1. Each dot represents the mean amount of money donated to the group investment (dollars). Error bars are +/− SE.

To test our theoretically driven hypothesis of a linear increase from the control to the shared goal condition, we conducted a Jonckheere-Terpstra test. We found that a significant linear trend fitted the data: *J* = 2242, *z* = −2.03, *p* = .04, (see Figure 1). The highest mean rank contribution was found for the synchrony plus shared goal condition (*Mdn* = $5.00), followed by the synchrony condition (*Mdn* = $4.50), the passive condition (*Mdn* = $3.25), and the smallest contribution was found in the asynchrony condition (*Mdn* = $2.75). Examining the overall improvement of shared intentionality to synchrony, Mann-Whitney *U* tests found that the level of investment in the shared goal condition was significantly higher than the asynchrony condition, *U = *277.50, *z* = −2.47, *p = *.01, *r = *.32; marginally significantly higher that the passive condition, *U = *329.50, *z* = −1.68, *p = *.09, *r = *.22, but was not significantly higher than the synchrony condition, *U = *395.00, *z* = −.66, *p = *.51, *r = *.09, (see Table 1).

**Table 1:** Means of prosocial outcome variables for Experiment 1 (standard deviations are in parenthesis).

	Shared goal	Synchrony	Asynchrony	Passive
Cooperation^a^	3.88 (1.59)	3.53(1.76), *r* = .09	2.87(1.72), *r* = .32*	3.22 (1.65), *r* = .22
Interdependent self-construal	3.69(1.71)	2.47(1.25), *d* = .81**	2.47(1.43), *d* = .77**	2.28(1.25), *d* = .94**
Entitativity	4.90(1.45)	4.78(1.80), *d* = .07	4.13(1.71), *d* = .49	3.41(1.96), *d* = .94**
Trust	4.48(1.62)	4.30(1.37), *d* = .12	4.23(1.45), *d* = .16	3.59(1.21), *d* = .62*
Perceived similarity	4.24(1.66)	3.77(1.52), *d* = .30	3.80(1.47), *d* = .28	3.34(1.32), *d* = .60*

a Effect sizes and significance values are based on the non-parametric Mann-Whitney tests.

* *p*<.05,

** *p*<.01.

Effect sizes and significance levels are in comparison to the shared goal condition.

**Figure 2:**
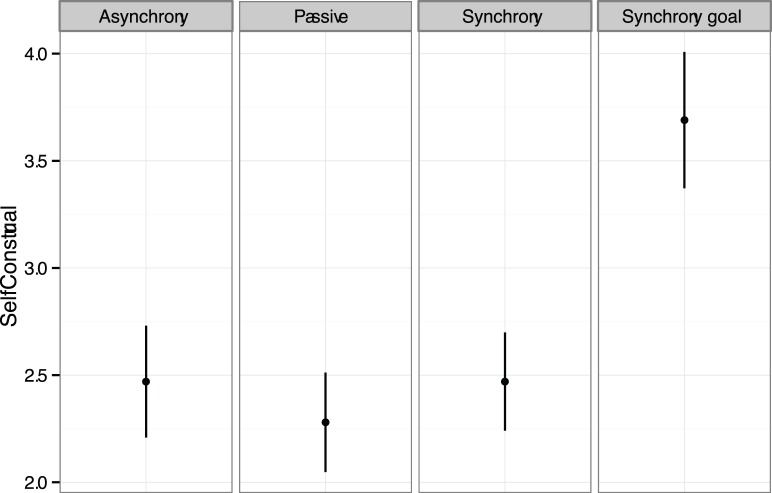
Interdependent self-construal across experimental conditions for Experiment 1. Each dot represents the mean rating for interdependent self-construal. Error bars are +/− SE.

One-way ANOVAs were performed for each of the self-report measures with movement condition as the independent variable. There was a significant difference in interdependent self-construal, *F*(3,114) = 6.06, *p* = .01, *η_p_* ^2^ = .14 with the shared goal condition producing significantly higher (*p*s<.01) interdependent self-construal than in each of the other three conditions (See Figure 2). A main effect was found for entitativity, *F*(3,114) = 4.51, *p* = .01, *η_p_* ^2^ = .11. A polynomial contrast analysis showed a significant linear trend, *F*(1, 114) = 12.94, *p*<.01, with the shared goal condition producing the highest mean level of entitativity, then the synchrony condition, then the asynchrony condition, and lastly the passive condition. Although the shared goal condition produced the highest mean level of entitativity, it was only significantly greater than the control condition. (Note that for those dependent variables where intra-class correlations suggested possible violation of independence of data within groups, we also conducted multi-level analyses [56]. Only for entitativity in Experiment 1 did this produce any substantial change in the results with the main effect of movement becoming marginally significant, *F*(3,32.07) = 2.63, *p* = .07).

Although the overall ANOVAs did not yield significant differences in responses for perceived similarity, *F*(3, 114) = 3.89, *p* = .16, or trust, *F*(3, 114) = 4.44, *p* = .09, both measures showed significant linear contrasts, such that the shared goal condition had the highest mean followed by the standard synchrony condition and asynchrony condition with the passive condition showing the lowest mean, *F*(1, 114) = 4.57, *p* = .04, and *F*(1, 114) = 5.48, p = .02, for perceived similarity and trust, respectively. No significant differences were found for perceived difficulty across the four conditions.

Entitativity and perceived similarity were not significantly correlated with the amount of money participant’s placed into the group investment (*r* = .13, *p* = .18, and *r* = .06, *p* = .55, respectively). Interdependent self-construal was marginally significantly correlated with the money placed into the group investment (*r* = .18, *p* = .05), and trust was significantly related to investment (*r* = .41, *p*<.01).

## Discussion Experiment 1

Shared intentionality combined with synchrony produced the highest level of cooperation and self-reported prosociality across all measures. Although the linear trend for cooperation in the public goods game was significant across the four conditions, the shared goal condition was only significantly greater than the asynchrony condition. However, the significant negative skew in the shared goal condition, with the majority of participants (55%) choosing the full $5, suggests that this cooperation measure may have been influenced by a ceiling effect. In line with expectations, we observed substantive differences across the experimental conditions for interdependent self-construal. Participants in the shared goal condition felt significantly closer to their fellow performance group members than compared to all of the other conditions, suggesting that working together to create synchrony results in participants seeing their self as more integrated with the group. This finding is important because it supports the idea that shared intentionality might be an important factor in producing the cooperative effects of collective music and dance.

## Experiment 2

To further explore the effect of shared intentionality combined with synchrony, we conducted a second study comparing the cooperative effects of creating synchrony through shared intentionality to the cooperative effects of creating asynchrony through shared intentionality. Additionally, to better understand whether synchrony extends to modalities beyond movement, Experiment 2 used a vocalisation paradigm.

In Experiment 1 we manipulated frequency-locked synchrony – participants in the asynchrony condition moved at different frequencies. However, participants in initial piloting were unable to *intentionally* chant at different rhythms in small groups (see [24] for reports of similar problems with drumming). Instead, we manipulated phase-locked synchrony, comparing in-phase to out-of-phase interaction. Several previous studies have examined the effect of phase on social cognition, finding that participants who moved in-phase remembered more information about another participant than participants who moved anti-phase (180° out of time) [57], [58]. To our knowledge, Experiment 2 is the first study to examine the influence of relative phase on cooperation.

Because our experiments involved groups larger than two, an anti-phase condition was inappropriate: at least two people would always end up vocalizing in-phase with each other as a by-product of moving anti-phase to a common target. To create a manipulation in which everybody would be out of phase with each other, participants were asked to vocalise one after another in a systematic rhythmic way – that is, participants were asked to vocalise sequentially.

We hypothesised that a greater proportion of participants vocalising in-phase with shared intentionality (synchrony condition) would cooperate than those who vocalised out-of-phase with shared intentionality (sequential condition). We also predicted that participants in the synchrony condition would report greater perceived similarity, interdependent self-construal, trust and entitativity than the sequential condition.

## Methods

### Participants

Participants were 27 volunteers assigned to groups of three (18 female; mean age = 22.23, range: 18–58 years) recruited through the same method as described for Experiment 1. All participants received a $5 reward for their involvement. Participants received an additional $7 or $10 depending on the outcome of the economic game.

### Procedure

#### Synchrony manipulation

We used a between-subjects design in which participants were randomly assigned to one of two vocalization conditions: synchrony and sequential. For the chanting activity, participants sat in chairs, in a row, in groups of three, and were asked to read out loud a list of 54 English words. Words were selected from the Affective Norms for English Words database [59] – a database of 1034 words with mean ratings of emotional valence from 1(negative) to 9 (positive) and emotional arousal from 1 (low) to 9 (high) for male raters and female raters. Emotionally neutral words were chosen, classified as words with a mean emotional valence rating between 4 and 6 and a mean emotional arousal rating less than 5 (for both males and female ratings). To facilitate synchronised pronunciations, only one-syllable words were selected (54 words in total). We generated twelve pages of emotionally neutral words in three columns, which were presented in a random order for participants to read. Participants read words from this list for 6 minutes. For the synchrony condition, all the participants in a group read the same words. In the sequential condition, each participant read the words from only one column.

In both conditions participants were explicitly instructed to act with a common goal. In the synchrony condition, participants were instructed that the goal of the activity was to speak the words in time with each other. For the sequential condition, participants were instructed that the goal of the activity was to speak the words out of time with each other – the leftmost participant was instructed to speak the leftmost words on the sheet, the middle participant the middle words, and the rightmost participant the rightmost words. A metronome beat was played through headphones for the first 20 seconds of the activity to help participants initially coordinate (60 bpm for the synchrony condition; 80 bpm for the sequential condition). These tempi were selected based on pre-tests that found that these speeds were the most comfortable for participants to complete each condition. To better motivate participants to follow instructions and to enhance a sense of a common fate, participants were also told that the experimenter would use a voice recorder to measure how accurately participants spoke in time or out of time with each other.

#### Cooperation measure

Experiment 2 used a coordination game with risk (Stag Hunt) rather than a public goods game. The decision to focus on risky coordination was driven by the following considerations. First, we were concerned that the unclear strategies underlying the public goods game might confound our cooperation measure. It has been demonstrated that experimental participants do not readily understand that defection always optimises financial returns in a standard public goods game [60], [61]. Second, we agree with those who argue that coordination games with risk are more common in human social life than prisoner’s dilemmas [62]–[67].

To make the risky coordination game both understandable and intuitive we used a binary choice according to which partners could select either option X or option Y (see Table 2). Option X returned a guaranteed reward of $7. Option Y returned $10 if the other partners opted for Y but returned $0 if at least one other partners opted for X. The optimal choice in this game reduced entirely to predicting what other partners would choose. Presumably all partners would value $10 over $7. Thus all participants had an incentive to cooperate, as there was no individual benefit from defecting when others donated. However, if even one player were to defect then the collective benefit would be lost. Thus if one were to defect then so too should everyone to obtain the safe defection payment and avoid the nil result from failed cooperation. The key difference between the prisoner’s dilemma (of which a public goods game is an example) and the stag hunt, is that in the prisoner’s dilemma defection is the dominant strategy, whereas in a Stag Hunt there are two equilibria: all cooperate and all defect. The particular payoff we used was based upon pre-testing using a sample of 21 participants of comparable demographics.

**Table 2:** Participants' choices for the stag hunt game used in Experiment 2 and 3.

Option X	Option Y
A guaranteed payment of **$7**, no matter what the other participants decide.	**$0** if any of the other participants choose X, **BUT** **$10** if all 3 participants choose Y.
	You will not know what the other participants will choose.

The same procedure as for the public goods game was used except that participants indicated if they would prefer option X or Y.

#### Post-activity questionnaire

Entitativity, perceived similarity, and trust were again measured on 7-point Likert scales but the scales were extended to include more items. Four items were used to measure entitativity (adapted from Lakens and Stel [52]): “How much did you experience a feeling of togetherness with the other participants?”, “How much did you feel you were on the same team with the other participants?”, “How much did you feel you and the other participants were a unit?”, “How much did you feel disconnected from the other participants?” (reverse coded), Cronbach alpha = .79. Two items measured perceived similarity: “how much did you feel similar to the other participants?” and “how much did you feel different to the other participants” (reverse coded), Cronbach alpha = .76. Included were two trust questions pertaining to the economic game: “How much did you trust the other participants going into the payment choice game?” and “How confident were you that the other group members would choose option Y (the $10 or $0 option)?”, Cronbach alpha = .67. The Inclusion of Self in Other (IOS) was used again to measure interdependent self-construal [55], but was modified with beginning of the scale extended with two further diagrams where the circles were separated at different distances [68] and with the amount of circle that overlapped following the scale developed by Swann and colleagues [69]. For manipulation checks, we created scales to measure perceived synchrony and perceived cooperation. We devised four items to quantify perceived synchrony, which were measured on 7-point Likert scales. These items were: “How much did you feel you were coordinated with the other participants?”, “How much did you feel the other participants and yourself spoke in unison with each other?”, “How much did you feel you were disjointed with the other participants?” (reverse coded), “How much did you feel the other participants and yourself spoke out-of-time with each other?” (reverse coded), Cronbach alpha = .72. One item was included to measure perceived cooperation: “How much did you feel you and the other participants cooperated during the task?”.

A priori, we expected that participants in the synchrony condition would perceive more synchrony than participants in the sequential condition; however, we expected that participants in both conditions would perceive similar levels of cooperation, given that both conditions required a shared intentional task.

Similar to Experiment 1, we asked participants to rate how difficult they found the speaking activity, and to rate how well they knew each other before the study.

## Results

The manipulation produced a significant difference in perceived synchrony with participants in the synchrony condition (*M* = 5.50, *SD* = 0.51) rating their condition as significantly more synchronous than participants in the sequential condition (*M* = 4.48, *SD* = 1.23), Welsh’s *t*(17.61) = 2.84, *p* = .01, *d* = 1.08. The sequential condition (*M* = 3.29, *SD* = 1.44) was rated as significantly more difficult than the synchrony condition (*M* = 2.08, *SD* = 1.32), *t*(25) = 2.27, *p* = .03, *d* = .88. However, both difficulty means were still on the lower half of the scale. This result suggests that participants did not find either condition too difficult. No significant difference between the conditions in how well participants knew each other beforehand was found, *t*(25) = 1.73, *p* = .10.

**Figure 3:**
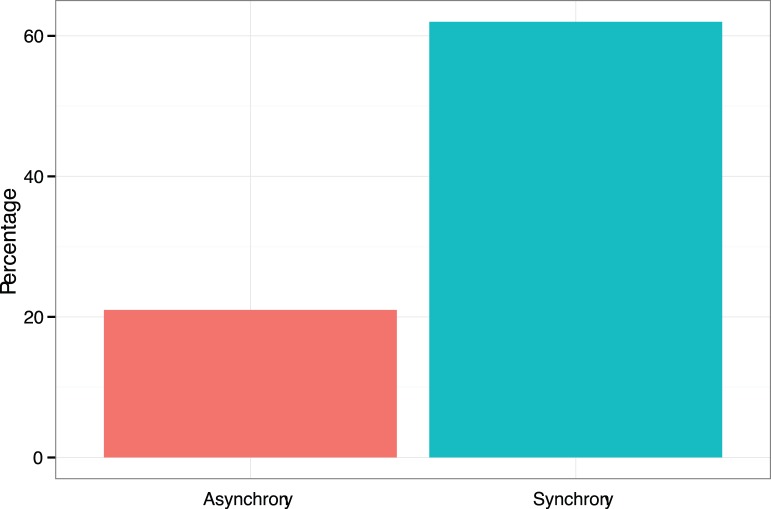
Cooperation across experimental conditions for Experiment 2. The percentage of participants choosing the cooperative Y option for each of the two vocalising conditions.

A Pearson chi square test found a significant association between the synchrony manipulation and the degree of cooperation, χ^2^(1,27) = 4.49, *p* = .03, with 62% of participants in the synchrony condition choosing the cooperative Y option compared to 21% in the sequential condition. Odds ratios indicated that the odds of a participant cooperating after vocalizing in synchrony were 5.87 times higher than after vocalizing sequentially. (See Figure 3).

No significant difference in perceived cooperation were observed, *t*(25) = 0.07, *p* = .95, perceived similarity, *t*(25) = 1.15, *p* = .26, entitativity, *t*(25) = 0.87, *p* = .39, interdependent self-construal, *t*(25) = 0.75, *p* = .26, and trust, *t*(25) = 0.33, *p* = .75. The only self-report variable that was correlated with cooperative behaviour was trust (*r* = .75, *p*<.01).

## Discussion Experiment 2

Experiment 2 revealed that participants who chanted in-phase cooperated more than participants who chanted sequentially. Synchrony boosted cooperation when shared intentionality was kept constant. This supports the claim that synchrony is an important factor in influencing cooperation in collective music performances. The lack of any significant differences between the synchrony and sequential conditions in the self-report measures was, however, in contrast to the findings of Experiment 1. In Experiment 1, we found significant differences between the shared goal condition and the comparison conditions in interdependent self-construal. One possibility for this discrepancy between Experiment 1 and Experiment 2 is that in Experiment 2 the comparison conditions did not involve shared intentionality. Therefore, it might have been the presence of shared intentionality rather than synchrony or the combination of synchrony and shared intentionality that boosted interdependent self-construal in Experiment 1. The addition of shared intentionality in the control condition in Experiment 2 might have also boosted self-reported interdependent self-construal, resulting in no difference between the conditions. Another explanation for this discrepancy is that participants in the sequential chanting condition used in Experiment 2 were still synchronous to some degree. Although not in phase-locked synchrony, participants in the sequential condition were nevertheless entrained to a common underlying beat and moved at the same frequency, i.e., participants were in *frequency-locked synchrony*. In support of this idea, participants in the sequential condition still rated their movement above the midpoint of the perceived synchrony scale (a mean of 4.48 on a scale of 1 to 7). If such an explanation was plausible, we would expect that intentionally chanting at different speeds (i.e. without entrainment) would have led to differences in self-report measures.

To better assess the interaction of synchrony and shared intentionality on cooperative behaviour and self-report measures, it is important to investigate the effects of manipulations that vary both synchrony and shared intentionality. This was the objective of Experiment 3, which included both an asynchrony condition and a sequential condition to also examine the relative importance of phase-relationship in producing the synchrony-cooperation effects observed in Experiments 1 and 2. Given the difficulties in creating asynchronous chanting, Experiment 3 employed body movement manipulations of synchrony.

## Experiment 3

Experiment 3 asked three questions. First we asked: does synchrony interact with shared intentionality to increase prosociality? To help answer this question, synchrony was manipulated with either a shared goal or an individual goal. The co-occurrence of synchrony and shared intentionality in naturally occurring music, dance, and traditional rituals led us to hypothesise that there would be a significant interaction between synchrony and shared intentionality. Specifically, we predicted that the difference in cooperation between the synchrony and control conditions should be greater when shared intentionality is used to create synchrony than when only an individual goal is used to create incidental synchrony. Furthermore, synchrony evoked in the context of shared goals should be associated with greater levels of cooperation than synchrony evoked in the context of individualistic goals.

**Figure 4:**
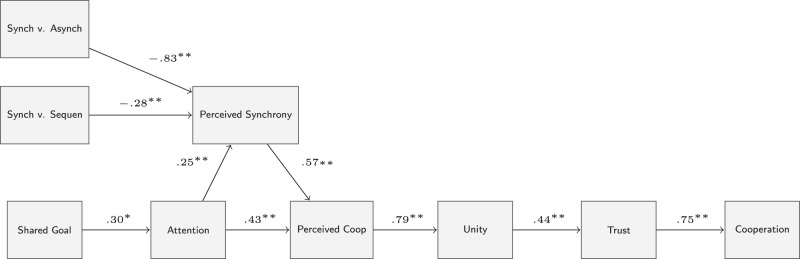
The reinforcement of cooperation model. (1) Movement manipulation: the creation of synchrony (synch) leads to greater perceived synchrony amongst the performers compared to asynchronous (asynch) or sequential (sequen) movement. (2) Goal manipulation: having a shared goal to coordinate movement in a certain way results in greater attention to the other participants’ movements. When a shared goal and synchrony are combined, this further strengthens perceived synchrony. Perceiving synchrony when there is a shared goal to produce synchrony provides immediate feedback of successful cooperation in performing the synchronous task together so increasing perceived cooperation. Increased feelings of successful cooperation lead to perceptions of social unity, from which participants feel greater trust and confidence that their fellow participants will cooperate in the future. Greater trust leads to greater cooperation in future interactions. Numbers above arrows represent standardised coefficients (* *p*<.05; ** *p*<.01).

Second we asked: what psychological processes support synchrony-cooperation effects? To help answer this question, we tested a theoretical model inspired by Hagen and Bryant’s [70] theory that music and dance signal a group’s cooperative ability to other groups. However, instead of signalling cooperative ability to other groups, we hypothesised that synchrony might serve to reinforce cooperation for the interacting group. According to our *reinforcement of cooperation model*, perceiving synchrony when there is a shared goal to produce synchrony provides immediate feedback of successful cooperation in performing the synchronous task together. With each iteration of a synchronous rhythmic action, fresh evidence of group cooperation is produced so further reinforcing the feeling that the group is successfully cooperating together. Increased feelings of successful cooperation, in turn, leads to perceptions of perceived similarity, entitativity, and interdependent self-construal, from which participants feel greater trust and confidence that their fellow participants will cooperate in the future. Greater trust in turns leads to greater cooperation in future interactions. This mechanism may be further strengthened through joint attention – to create synchrony through shared intentionality requires participants pay careful attention to each other so creating a greater awareness of synchrony, and therefore further strengthening perceived cooperation, offering additional boosts to cooperative prediction (see Figure 4).

Third we asked: do the prosocial effects of frequency-locked synchrony differ from those of phase-locked synchrony? This question is relevant to synchronous movements in natural human ecologies because many types of dance and music require performers to purposefully move out of-phase with each other, whilst still entrained to a common underlying beat. To help answer this question, we used two control conditions, one in which participants were entrained to a common beat but moved out of phase with each other (sequential condition); and a second in which participants moved at different speeds and thus were neither phase-locked nor frequency-locked to each other’s movements (asynchrony condition). The significant difference in prosociality in Experiment 2 between the synchrony and sequential conditions suggests that being in-phase does increase cooperation compared to being out-of-phase but entrained. We therefore predicted that the synchrony condition would lead to significantly greater cooperation than both the sequential and asynchrony condition. However, based on the observation that many forms of music and dance involve complex phase relations, we also predicted that moving sequentially would result in greater cooperation than moving in asynchrony.

## Methods

### Participants

Participants were 86 volunteers in groups of three recruited through the same method as described for Experiment 1 (61 female; mean age = 23.71, range: 16–60 years).

### Procedure

We investigated the interaction of shared goals and synchrony using a 3 (movement) x 2 (goal) factorial design. The three levels of the movement manipulation were: (1) synchrony – participants moved the same way at the same time; (2) sequential – participants moved at different times but still entrained to the same beat; and (3) asynchrony – participants moved at different speeds resulting in them moving out of time with each other. The two levels of the goal manipulation were: (1) shared goal – participants worked together to complete a shared goal (that is they used shared intentionality); and (2) individual goal – participants were told to move in time with the metronome beat coming through their headphones. (See Table 3 for a summary of the conditions).

**Table 3:** Summary of the goals participants were given for each of the six conditions used in Experiment 3.

	Movement condition		
Goal condition	Synchrony	Sequential	Asynchrony
Group Goal	Work together to move in time	Work together to move sequentially	Work together to move out of time
Individual Goal	Move in time with beat	Move in time with beat	Move in time with beat

### Synchrony Manipulation

To accentuate goals, and to provide a more precise method to measure the relative timing of participants’ behaviour, foot-pedals where introduced that relayed information about the timing of participants’ movements to a computer. Instead of the three different movements, participants were asked to rhythmically step on the foot-pedals with alternating feet for the full six minutes. Participants were also instructed to move their left arm forward with their left leg, and their right arm forward with their right leg.

For the individual goal conditions, participants heard the metronome beat for the full length of the movement activity and were told that the goal of the activity was: “to move in time with the rhythmic pulse being played through your headphones”. In the synchrony individual goal condition participants heard the same metronome beat (55 bpm). In the asynchrony individual goal condition each participant was played a metronome beat at a different speed (45 bpm, 55 bpm, 65 bpm). For the sequential individual goal condition, a slightly different metronome beat was designed (played at 45 bpm) with the first two beats presented as a low pitched drum, the next two beats as a mid-tone drum, and the last two beats as a high pitched drum. The left-most participants moved their left feet and arms forward and back on the first 2 beats. Middle participants moved their left feet and arms forward and back on the next 2 beats; right-most participants moved their left feet and arms forward and back on the last two beats.

For the group goal conditions, we created shared intentionality by providing participants with slightly different instructions. In the synchrony group goal condition participants were told that the goal of the activity was: “to move like this in time with each other; this means that you are consistently pressing the pedal at the same time as each other, and moving at the same speed.” Participants in the sequential-group goal conditions were told that the goal of the activity was: “to move like this sequentially and at the same speed as each other”. Participants in the asynchrony-group goal conditions were told that the goal of the activity was: “to move like this out of time with each other; this means that you are *not* consistently pressing the pedals at the same time as another, but will be moving at different speeds”. To help participants coordinate at the beginning of each condition, the appropriate metronome beat for the condition was played for the first 20 seconds of their movement. Participants were told that after 20 seconds the pulse will stop and they will need to work together to stay in time/move sequentially/stay out of time. To facilitate out-of-time movements in the asynchrony conditions, left-most participants who initially heard the slowest 45 bpm metronome were told to slow the speed of their movements down a little if they found themselves moving in time, whereas rightmost participants who initially heard the 65 bpm metronome were told to speed up if they found themselves moving in time.

Goal salience was accentuated both by informing participants that foot-pedal activations were being measured and by their performance being video recorded. Additionally, participants in the individual goal conditions were instructed that “it is important that you do the best you can to keep in time with the pulse” whereas participants in the group goal conditions were instructed that “it is important that you work together to keep in time/keep coordinated/keep out of time with each other.”

### Cooperation Measure and Post-activity Questionnaire

Experiment 3 used the same stag-hunt protocol used in Experiment 2. The same post-activity questionnaire from Experiment 2 was also used with measures for perceived synchrony (Cronbach alpha = .83), similarity (Cronbach alpha = .56), entitativity (Cronbach alpha = .77), trust (Cronbach alpha = .81), and the one-item measures of cooperation and interdependent self-construal. Because similarity, entitativity, and interdependent self-construal tapped into a similar underlying construct of how the self is seen in relation to others, we examined the possibility of combining these measures into a single factor. Perceived similarity and interdependent self-construal were both strongly positively correlated with entitativity (*r = *.63, *p*<.01 and *r* = .59, *p*<.01 for perceived similarity and interdependent self-construal with entitativity respectively). A principal components factor analysis on the seven items (4 entitativity items, 2 similarity items, 1 self-construal item) with varimax rotation, using Kaiser’s criterion of eigenvalues greater than 1, confirmed that there was only one factor. This single factor, which we termed *social unity*, explained 52.86% of the variance in the items. The internal consistency reliability of this social unity measure was also good (Cronbach alpha = .82).

Four items were also used to measure the amount of attention paid to the other participants: “How much did you pay attention to the other participants?”, “How much did you find the other participants aided in doing the activity?”, “How much did you try to ignore the other participants?” (reverse coded), “How much did you find the other participants distracting?” (reverse coded), (Cronbach alpha = .72).

## Results

Two participants were removed who were in the same group and reported being in a committed romantic relationship together. Two participants were also removed because they spent nearly 3 times as long as other participants completing the questionnaire, suggesting their level of English was not adequate. No significant main effects or interactions in how well participants knew each other beforehand were found, *F*_max_ = 1.69, *p* = .19.

### Manipulation Check

The timing of the foot-pedal presses, self-reported perceived synchrony, perceived cooperation, and perceived difficulty were analysed to check that the synchrony and goal manipulations worked (see Data S1).

### Cooperation

**Figure 5:**
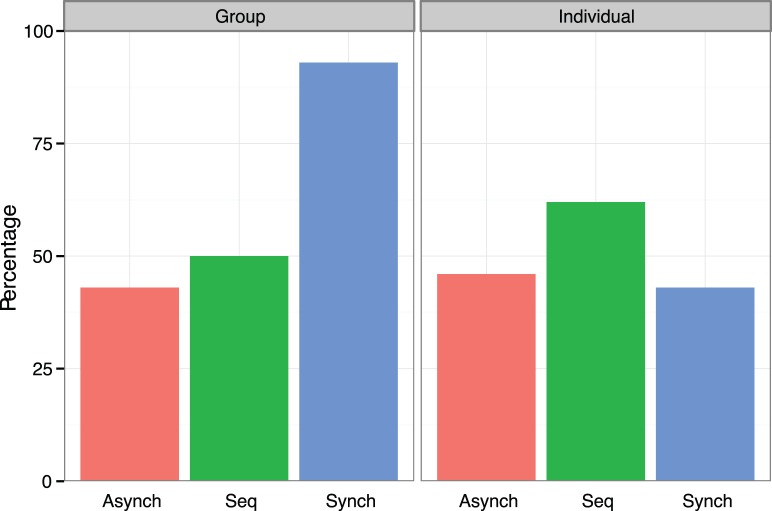
Cooperation across experimental conditions for Experiment 2. The percentage of participants choosing the cooperative Y option for when synchronous, sequential or asynchronous movement is created through a group goal or an individual goal.

To examine the effect of the manipulations on cooperation, a three-way hierarchical log-linear analysis with backward elimination was conducted with movement (3 levels), goal (2 levels), and cooperative behaviour (2 levels). A significant Movement x Goal x Behaviour interaction was found, χ^2^(2,82) = 7.94, *p* = .02. Follow-up chi-square tests were used to examine this interaction. These tests revealed a significant difference between the group goal conditions, χ^2^(2,42) = 8.68, *p* = .01. The synchrony group goal condition was significantly different from both the sequential group goal condition, χ^2^(1,28) = 6.30, *p* = .01, and the asynchrony group goal condition, χ^2^(1,28) = 8.02, *p* = .01. No significant difference existed between the sequential and asynchrony condition, χ^2^(1,28) = .14, *p* = .71. Odds ratios tests indicated that the odds of a participant cooperating after moving in synchrony was 13.00 times higher than after moving sequentially and 17.33 times higher than after moving asynchronously. No significant differences were found in the individual goal condition, χ^2^ (2,40) = 1.06, *p* = .59. Comparisons between the individual goal conditions indicated that the odds of a participant cooperating after moving in synchrony was 2.13 times lower after moving sequentially and 1.17 times lower than after moving asynchronously. As shown in Figure 5, the interaction between movement and goals on cooperation was primarily driven by the synchrony group goal condition, where 93% of participants chose the cooperative Y option compared with between 43%–62% who opted for the cooperative Y in the other conditions. These results indicate that the combination of shared intentionality and synchrony is important.

### Post-activity Questionnaire

To examine the effects our manipulations had on psychological states, we ran a 3 (movement) x2 (goal) factorial ANOVA for social unity, trust and attention. There was no significant interaction or main effect of goal on social unity (*F*_max_ = 1.33, *p* = .27). However we found a significant main effect of movement on social unity, *F*(2,76) = 6.15, *p*<.01, *η_p_* ^2^ = 0.14. Gabriel post-hoc tests revealed that the synchrony condition (*M* = 4.52, *SD* = 0.97) had a significantly higher mean for social unity than the asynchrony condition (*M* = 3.58, *SD* = 1.22; *p*<.01, *d* = 0.85), but not the sequential condition (*M* = 4.13, *SD* = 1.07; *p* = .45, *d* = 0.38). There was also no significant difference in social unity between the sequential and asynchrony conditions (*p* = .12, *d* = 0.48). Importantly, social unity was significantly positively correlated with participants’ choice of the cooperative Y option (*r*_pb_ = .32, *p*<.01).

Comparisons of attention directed towards other participants revealed a significant main effect for synchrony, *F*(2,76) = 6.46, *p*<.01, *η*_p_^2^ = 0.15, and a significant main effect for goal, *F*(2,76) = 6.15, *p* = . 02, *η*_p_^2^ = 0.08, but no significant interaction, *F*(2,76) = 1.80, *p* = .17, *η*_p_^2^ = 0.05. Exploring the main effect of synchrony, Gabriel post-hoc tests revealed that participants paid more attention to their fellow participants in the synchrony condition (*M = *5.26, *SD* = 1.04) than in the asynchrony condition (*M* = 4.18, *SD* = 1.46; *p*<.01, *d* = 85). The sequential condition (*M* = 4.98, *SD* = 1.17) was also associated with significantly higher attention than the asynchrony condition (*p* = .04, *d* = 0.60), but did not differ from the synchrony condition (*p* = .76, *d* = 0.25). Explorations of the main effect of goal revealed that the group goal condition (*M* = 5.12, *SD* = 1.27) had a significantly higher mean than the individual goal condition (*M* = 4.49, *SD* = 1.27; *d* = 0.50).

No main effects or interactions were found for trust, *F*_max_ = 1.00, *p* = .37.

### Path Analysis

The reinforcement of cooperation model was tested using MPlus [71]. Because the synchrony manipulation had three levels, two dummy codes were used for the path analysis. Our main interest was in the effect of synchrony compared to the sequential and asynchrony conditions. Thus one dummy variable was used when comparing the synchrony condition with the asynchrony condition and the other dummy variable was used when comparing the synchrony condition with the sequential condition. The model provided a very good fit: χ2 (24) = 32.06, p = .13; CFI = .94, RMSEA = .06, WRMR = .71 (see Figure 4 for standardised coefficients). Yu and Muthén suggest CFI>.95, RMSEA<.06, WRMR<.90 as cut off values for good models when the outcome variable is categorical (cited in [72]).

We also tested an alternative model in which psychological reactions are consequences of behaviour: greater social unity is produced from acting cooperatively in the economic game. This is in line with self-perception theory [73] that states that people derive their psychological states based on their observation of behaviour. Importantly, this reversed causal model did not fit the data well: χ^2^ (22) = 89.28, *p*<.01; CFI = .73, RMSEA = .19, SRMR = .17. This shows that the reinforcement of cooperation model is robust against plausible alternative models.

## Discussion Experiment 3

Experiment 3 found that when participants worked together to create synchrony nearly all participants opted for the cooperative option, despite its risks. Working together to move out of time did not appear to significantly influence cooperation, nor did moving in synchrony without shared intentionality. The lack of any significant difference between the movement conditions in the individual goal condition was interesting, given previous studies showing that synchrony manipulated through entrainment to a recorded beat increased prosocial behaviour [34], [37], (but see [74], [75]). Speculating, it is possible that instructions to act in synchrony tend to evoke shared-intentionality even when participants are not explicitly instructed to follow a shared goal. In the individual goal condition where we explicitly emphasised an individual goal, this lead to a reduction of any implicit shared intentionality so reducing any synchrony-cooperation effect. In the group goal condition where we explicitly emphasised a group goal, this lead to an increase in shared intentionality so increasing the synchrony-cooperation effect. Collectively these findings suggest that it is the interaction of group synchrony with shared goals that leads to increased prosocial behaviours.

The path analysis supported the reinforcement of cooperation model that we proposed to explain how synchrony and shared intentionality may combine to increase cooperation. In this model, perceived synchrony when framed by a collective goal to generate synchrony provides evidence of successful cooperation Evidence of successful cooperation leads to feelings of unity, and trust, which in turn supports subsequent cooperative behaviours. Although the path analysis shows that our data is *consistent* with our proposed theoretical model, we can only claim support for a causal relation between the independent variables of our design (movement and goal) and our dependent variables (cooperative behaviour and self-report data). Evidence of the causal relation between the dependent variables in our model will need to be subsequently investigated.

The third aim of Experiment 3 was to assess the relative importance of phase-locked synchrony versus frequency-locked synchrony. For cooperative behaviour, the synchrony condition resulted in significantly more cooperation than were observed in both the asynchrony and sequential conditions. This suggests that being in-phase is important to produce an increase in cooperation. Although descriptive statistics indicate that the proportion of participants who choose the cooperative option was greater in the sequential condition than the asynchrony condition, this difference was not significant. This would seem to indicate that it is phase-locked synchrony rather than frequency-locked synchrony that fosters cooperation.

Our finding of no significant difference between the sequential and asynchrony conditions may appear surprising given that out-of-phase frequency-locked synchrony is common across cultures. It is possible that our manipulation of frequency-locked synchrony did not capture the complex inter-personal phase patterning that is commonly found in music and dance. For example, in the sequential condition, participants performed and then stood passively while the other group members performed. This may have made people more self-aware as only one participant was performing at a time, so increasing their feelings of being an individual. An increase in cooperation may have been found if participants were continuously performing out of phase with each other – a question that could be explored in future research.

### Conclusion

The results of the three experiments reported above indicate that synchrony promotes cooperation more powerfully when it is framed as a collective goal. We found that synchrony combined with shared intentionality leads to greater cooperation than synchrony without shared intentionality or shared intentionality combined with asynchronous movement or vocalising. Our study offers the first demonstration that synchrony interacts with explicitly shared goals to support cooperative interactions. Additionally, we present and empirically assess a plausible psychological model for the proximate cognitive processes that underpin heightened prosociality, when synchrony and collective goals combine.

### Limitations

Our primary aim of these three experiments was to examine the causal relationship between synchrony, shared intentionality and cooperation. To create strong internal validity to examine this question, our artificial creation of collective song and dance deviated from that commonly found in human ecologies. For example, most natural expressions of music and dance last considerably longer than the six minutes used in our experiments; are performed by groups of people who know each other well usually with an existing group identity; and often involve group identifiers such as similar dress, a shared cultural meaning of the dance or music, and lyrics that prime unique attributes of the group [76]–[78]. It is unclear how these factors may interact with shared intentionality and synchrony to affect cooperative outcomes. It would be important in future research to investigate how these different factors combine in natural ecologies [79].

### Importance of Findings

Synchrony involves the temporal and often spatial matching of behaviour to another individual. One theory that has been proposed for the cooperative effects of synchrony (along with related phenomena such as mimicry) is that the joint recruitment of motor and perceptual systems needed for such imitation results in a blurring of the self with another [27], [30], [80]–[82]. An important contribution of this study is the observation that in group synchrony, such low-level action and perception systems combine with higher level intentional systems to evoke especially powerful cooperative responses. We have shown it may be an alignment of ideals in concert with an alignment of bodies that amplifies cooperative responses. This finding contributes to an increasing chorus among cognitive neuroscientists emphasizing the importance of investigating relationships between implicit and explicit cognition [83], [84].

We also believe that our study holds interest for evolutionary scholars. It has long been conjectured that collective music and dance are bio-cultural adaptations for cooperation [6], [9], [12], [85]–[87]. Such functionality could explain the universality and retention of music and dance across all known human societies, present and past, despite associated energy, resource, and opportunity costs [10]. In line with past research [37], our finding that greater cooperation is associated with collective synchrony offer support for these adaptationist intuitions. We have taken these findings further by showing that the framing of coordinated behaviour with purposes that transcend personal interests produces an even more powerful cooperative response than synchronous interaction in isolation from collective goals.

## Supporting Information

Data S1 (DOC)

## References

[1] Blacking J (1973) How musical is man. Seattle: University of Washington Press.

[2] Brown D (1991) Human universals. New York: McGraw-Hill.

[3] Lomax A (1968) Folk song style and culture. New Brunswick, N.J.: Transaction Books.

[4] Adler DS (2009) The earliest musical tradition. Nature 460: 695–696 doi:10.1038/460695a 19661905 10.1038/460695a

[5] Conard NJ, Malina M, Munzel SC (2009) New flutes document the earliest musical tradition in southwestern Germany. Nature 460: 737–740 doi:10.1038/nature08169 19553935 10.1038/nature08169

[6] Bispham J (2010) Music’s “design features”: Musical motivation, musical pulse, and musical pitch. Musicae Scientiae: 29–44.

[7] Brown S (2000) Evolutionary models of music: From sexual selection to group selection. In: Tonneau F, Thompson NS, editors. Perspectives in ethology 13: Behavior, evolution and culture. New York: Plenum Publishers. 231–281.

[8] Cross I (2001) Music, cognition, culture, and evolution. Annals of the New York Academy of sciences: 28–42. 10.1111/j.1749-6632.2001.tb05723.x 11458835

[9] Freeman WJ (2000) A neurobiological role of music in social bonding. In: Wallin NL, Merker B, Brown S, editors. The origins of music. Cambridge, MA: MIT Press. 411–424.

[10] Huron D (2001) Is music an evolutionary adaptation? Annals of the New York Academy of sciences 930: 43–61. 11458859 10.1111/j.1749-6632.2001.tb05724.x

[11] Levitin DJ (2008) World in six songs: How the musical brain created human nature. New York: Dutton.

[12] Mithen S (2005) The singing Neanderthals: The origins of music, language, mind and body. London: Orion.

[13] Roederer J (1984) The search for a survival value of music. Music Perception 1: 350–356.

[14] Durkheim E (1965) The elementary forms of the religious life (J. W. Swain, Trans.). New York: Free Press. (Original work published 1915). Available: http://www.archive.org/stream/elementaryformso00durk#page/n1/mode/2up. Accessed 2011 Jun 25.

[15] Ellis H (1923) The dance of life. New York: Houghton Mifflin.

[16] Grosse E (1897) The beginnings of art. New York: D. Appleton and Company. Available: http://archive.org/stream/beginningsofart00grosuoft#page/n7/mode/2up. Accessed 2012 May 12.

[17] Radcliffe-Brown AR (1948) The Andaman Islanders. Glencoe: The Free Press.

[18] Lange R (1975) The nature of dance: An anthropological perspective. London: Macdonald and Evans.

[19] Spencer P (1985) Interpretations of the dance in anthropology. In: Spencer P, editor. Society and the dance: the social anthropology of process and performance. Cambridge, U.K: Cambridge University Press. 1–46.

[20] Gorer G (1972) Function of dance forms in primitive African communities. In: Boas F, editor. The function of dance in human society. New York: Dance Horizons. 21–40.

[21] Kuper H (1947) An African aristocracy: Rank among the Swazi of Bechuanaland. London: Oxford University Press.

[22] McNeill WH (1995) Keeping together in time: Dance and drill in human history. Cambridge, MA: Harvard University Press.

[23] Ehrenreich B (2006) Dancing in the streets: A history of collective joy. New York: Metropolitan.

[24] Kirschner S, Tomasello M (2010) Joint music making promotes prosocial behavior in 4-year-old children. Evolution and Human Behavior 31: 354–364.

[25] Anshel A, Kipper DA (1988) The influence of group singing on trust and cooperation. Journal of Music Therapy 25: 145–155.

[26] Clayton M, Sager R, Will U (2004) In time with the music: The concept of entrainment and its significance for ethnomusicology. CounterPoint 1: 1–45.

[27] Benzon W (2001) Beethoven’s anvil: Music in mind and culture. New York: Basic Books.

[28] Bernieri FJ (1988) Coordinated movement and rapport in teacher-student interactions. Journal of Nonverbal Behavior 12: 120–138.

[29] Haidt J, Seder JP, Kesebir S (2008) Hive psychology, happiness, and public policy. Journal of Legal Studies 37: S133–S156.

[30] Hove MJ, Risen JL (2009) It’s all in the timing: Interpersonal synchrony increases affiliation. Social Cognition 27: 949–960.

[31] Kendon A (1970) Movement coordination in social interaction: Some examples described. Acta Psychologica 32: 101–125. 5444439

[32] Kokal I, Engel A, Kirschner S, Keysers C (2011) Synchronized drumming enhances activity in the caudate and facilitates prosocial commitment - if the rhythm comes easily. PLoS ONE 6: e27272. Available: http://dx.plos.org/10.1371/journal.pone.0027272. Accessed 2012 Nov 18. 10.1371/journal.pone.0027272 PMC3217964 22110623

[33] Miles LK, Griffiths JL, Richardson MJ, Macrae CN (2010) Too late to coordinate: Contextual influences on behavioral synchrony. European Journal of Social Psychology 40: 52–60 doi:10.1002/ejsp.721

[34] Valdesolo P, DeSteno D (2011) Synchrony and the social tuning of compassion. Emotion 11: 262–266. 21500895 10.1037/a0021302

[35] Valdesolo P, Ouyang J, DeSteno D (2010) The rhythm of joint action: Synchrony promotes cooperative ability. Journal of Experimental Social Psychology 46: 693–695.

[36] Warner RM, Malloy D, Schneider K, Knoth R, Wilder B (1987) Rhythmic organization of social interaction and observer ratings of positive affect and involvement. Journal of Nonverbal Behavior 11: 57–74.

[37] Wiltermuth SS, Heath C (2009) Synchrony and cooperation. Psychological Science 20: 1–5 doi:10.1111/j.1467-9280.2008.02253.x 19152536 10.1111/j.1467-9280.2008.02253.x

[38] Cohen EEA, Ejsmond-Frey R, Knight N, Dunbar RIM (2010) Rowers’ high: behavioural synchrony is correlated with elevated pain theshholds. Biology Letters 6: 106–108. 19755532 10.1098/rsbl.2009.0670 PMC2817271

[39] Condon WS, Ogston WD (1967) A segmentation of behavior. Journal of Psychiatric Research 5: 221–235.

[40] Dittmann AT, Llewellyn LG (1969) Body movement and speech rhythm in social conversation. Journal of Personality and Social Psychology 11: 98–106. 5778352 10.1037/h0027035

[41] Hadar U, Steiner TJ, Rose FC (1985) Head movement during listening turns in conversation. Journal of Nonverbal Behavior 9: 214–228.

[42] Rutter DR, Stephenson GM (1977) The role of visual communication in synchronizing conversation. European Journal of Social Psychology 7: 29–37.

[43] Shockley K, Santana MV, Fowler CA (2003) Mutual interpersonal postural constraints are involved in cooperative conversation. Journal of Experimental Psychology-Human Perception and Performance 29: 326–332. 12760618 10.1037/0096-1523.29.2.326

[44] Van Ulzen NR, Lamoth CJC, Daffertshofer A, Semin GR, Beek PJ (2008) Characteristics of instructed and uninstructed interpersonal coordination while walking side-by-side. Neuroscience Letters 432: 88–93 doi:10.1016/j.neulet.2007.11.070 18242846 10.1016/j.neulet.2007.11.070

[45] Zivotofsky AZ, Hausdorff JM (2007) The sensory feedback mechanisms enabling couples to walk synchronously: An initial investigation. Journal of Neuroengineering and Rehabilitation 4: 5 doi:28 10.1186/1743-0003-4-28 17686150 10.1186/1743-0003-4-28 PMC1973071

[46] Tomasello M, Carpenter M (2007) Shared intentionality. Developmental science 10: 121–125 doi:10.1111/j.1467-7687.2007.00573.x 17181709 10.1111/j.1467-7687.2007.00573.x

[47] Tomasello M, Carpenter M, Call J, Behne T, Moll H (2005) Understanding and sharing intentions: The origins of cultural cognition. Behavioral and Brain Sciences 28: 675–735. 16262930 10.1017/S0140525X05000129

[48] Campbell D (1958) Common fate, similarity, and other indices of the status of aggregates of persons as social entities. Behavioral Science 3: 14–25.

[49] Mitkidis P, Sørensen J, Nielbo KL, Andersen M, Lienard P (2013) Collective-goal ascription increases cooperation in humans. PloS one 8. doi:10.1371/journal.pone.0064776. 10.1371/journal.pone.0064776 PMC3660378 23705011

[50] Naruse K, Hirai T (2000) Effects of slow tempo exercise on respiration, heart rate, and mood state. Perceptual and Motor Skills 91: 729–740. 11153839 10.2466/pms.2000.91.3.729

[51] Lakens D (2010) Movement synchrony and perceived entitativity. Journal of Experimental Social Psychology 45: 701–708.

[52] Lakens D, Stel M (2011) If they move in sync, they must feel in sync: Movement synchrony leads to attributions of rapport and entitativity. Social Cognition 29: 1–14.

[53] Reddish P, Bulbulia J, Fischer R (2013) Does synchrony promote generalized prosociality? Religion, Brain and Behavior. doi:10.1080/2153599X.2013.764545

[54] Friedman D, Sunder S (1994) Experimental methods: A primer for economists. New York : Cambridge University Press.

[55] Aron A, Aron EN, Smollan D (1992) Inclusion of other in the self scale and the structure of interpersonal closeness. Journal of Personality and Social Psychology 63: 596–612.

[56] Kenny DA, Kashy DA, Cook WL (2006) Dyadic data analysis. New York: Guilford Publications.

[57] Macrae CN, Duffy OK, Miles LK, Lawrence J (2008) A case of hand waving: Action synchrony and person perception. Cognition 109: 152–156 doi:10.1016/j.cognition.2008.07.007 18755450 10.1016/j.cognition.2008.07.007

[58] Miles LK, Nind LK, Henderson Z, Macrae CN (2010) Moving memories: Behavioral synchrony and memory for self and others. Journal of Experimental Social Psychology 46: 457–460.

[59] Bradley MM, Lang PJ (1999) Affective norms for English words (ANEW): Instruction manual and affective ratings. Technical Report C-1. Gainesville, FL: The Center for Research in Psychophysiology, University of Florida.

[60] Andreoni J (1995) Cooperation in public-goods experiments: kindness or confusion? American Economic Review 85: 891–904.

[61] Houser D, Kurzban R (2002) Revisiting kindness and confusion in public goods experiments. American Economic Review 92: 1062–1069.

[62] Binmore K (1998) Game theory and the social contract: Just playing. Cambridge, MA: MIT Press.

[63] Bulbulia J (2012) Spreading order: religion, cooperative niche construction, and risky coordination problems. Biology and Philosophy 27: 1–27 doi:10.1007/s10539-011-9295-x 22207773 10.1007/s10539-011-9295-x PMC3223343

[64] Calcott B (2008) The other cooperation problem: generating benefit. Biology and Philosophy 23: 179–203.

[65] Schelling T (1960) The strategy of conflict. New York: Oxford University Press.

[66] Schelling T (1973) Hockey helmets, concealed weapons, and daylight saving. A study of binary choices with externalities. Journal of Conflict Resolution 17: 381–428.

[67] Skyrms B (2004) The stag hunt and the evolution of social structure. Cambridge, UK: Cambridge University Press.

[68] Schubert TW, Otten S (2002) Overlap of self, ingroup, and outgroup: Pictorial measures of self-categorization. Self and Identity 1: 353–376.

[69] Swann WB, Gomez A, Seyle DC, Morales JF, Huici C (2009) Identity fusion: The interplay of personal and social identities in extreme group behavior. Journal of Personality and Social Psychology 96: 995–1011 doi:10.1037/a0013668 19379032 10.1037/a0013668

[70] Hagen EH, Bryant GA (2003) Music and dance as a coalition signaling system. Human Nature 14: 21–51. 26189987 10.1007/s12110-003-1015-z

[71] Muthén LK, Muthén BO (2010) Mplus user’s guide. Version 6. Los Angeles, CA: Muthén & Muthén.

[72] Muthén BO (2004) Mplus technical appendices. Los Angeles, CA: Muthén & Muthén.

[73] Bem DJ (1967) Self-perception: An alternative interpretation of cognitive dissonance phenomena. Psychological Review 74: 183–200. 5342882 10.1037/h0024835

[74] Cohen E, Mundry R, Kirschner S (2013) Religion, synchrony, and cooperation. Religion, Brain & Behavior. doi:10.1080/2153599X.2012.741075.

[75] Schachner A, Garvin L (2010) Does synchrony really affect social variables? Effects on cooperation, conformity may not be robust. Poster presented at the International Conference on Music Perception and Cognition, Seattle, WA.

[76] Boer D, Fischer R (2012) Towards a holistic model of functions of music listening across cultures: A culturally decentred qualitative approach. Psychology of Music 40: 179–200 doi:10.1177/0305735610381885

[77] Giles H, Denes A, Hamilton DL, Hajda JM (2009) Striking a chord: A prelude to music and intergroup relations research. Group Processes & Intergroup Relations 12: 291–301 doi:10.1177/1368430209102840

[78] Hanna JL (1979) Movements toward understanding humans through the anthropological study of dance. Current Anthropology 20: 313–339.

[79] Fischer R, Callander R, Reddish P, Bulbulia J (2013) How do rituals affect cooperation? Human Nature 24: 115–125. 23666518 10.1007/s12110-013-9167-y

[80] Hove MJ (2008) Shared circuits, shared time, and interpersonal synchrony. Behavioral and Brain Sciences 31: 29–30 doi:10.1017/s0140525x07003202

[81] Smith ER (2008) An embodied account of self-other “overlap” and its effects. In: Semin GR, Smith ER, editors. Embodied grounding: Social, cognitive, affective, and neuroscientific approaches. New York: Cambridge University Press.

[82] Chartrand TL, Van Baaren R (2009) Human mimicry. In: M.P Zanna, editor. Advances in experimental social psychology. San Diego: Elsevier Academic Press, Vol. 41: 219–274.

[83] Knoblich G, Butterfill S, Sebanz N (2011) Psychological research on joint action: Theory and data. In: Ross B, editor. The psychology of learning and motivation. Burlington: Academic Press, Vol. 54: 59–101.

[84] Newell BR, Shanks DR (2013) Unconscious influences on decision making: A critical review. Behavioral and Brain Sciences. In press. 10.1017/S0140525X12003214 24461214

[85] Cross I (1999) Is music the most important thing we ever did? Music, development and evolution. In: Yi S, editor. Music, mind and science. Seoul: Seoul National University press. 10–39.

[86] Cross I (2009) The nature of music and its evolution. In: Hallam S, Cross I, Thaut MH, editors. The Oxford handbook of music psychology. New York: Oxford University Press. 3–13.

[87] Hauser MD, McDermott J (2003) The evolution of the music faculty: a comparative perspective. Nature Neuroscience 6: 663–668 doi:10.1038/nn1080 12830156 10.1038/nn1080

