# Supplementary material for: Let’s Dance Together: Synchrony, Shared Intentionality and Cooperation
Source: PLoS One. 2013 Aug 7;8(8):e71182. doi: 10.1371/journal.pone.0071182 (PMC3737148; doi:10.1371/journal.pone.0071182)
Supplement: Data S1 — (DOC) [file pone.0071182.s001.doc]

**Supplementary data: Manipulation checks for Experiment 3**

**Foot-pedal timing**

The timing of each foot-pedal press was recorded onto a computer to serve as an objective measure to check that the synchrony manipulation worked. Circular statistics [1] were conducted on this data examining the distribution of the phase difference between participants in a group. To calculate the phase differences for each participant, each foot-pedal press at time point *t* was matched up to the appropriate interval between foot-pedal presses (inter-stimulus interval [ISI]) for each of the other two participants in the group. This was done in pairs, such that each time point for participant A was matched onto the appropriate ISI for B and then the appropriate ISI for C and so on. The ISI interval was calculated by finding the foot-pedal presses of the matched participant that were just before and just after time point *t*. An asynchrony deviation was calculated by subtracting the earlier time point of the ISI from *t*. This asynchrony deviation was then converted into θ (in degrees) by multiplying it by 360° and then dividing by the ISI. For example, to find the relative phase match of participant A compared to participant B at a foot-pedal press at time *t* by participant A (t_A_), the following equation was used, where t_B1_ is the maximum time point of a foot-pedal press by participant B that is less than t_A_, and t_B2_ is the minimum time point of a foot-pedal press by participant B that is greater than t_A_:

For each participant, $\bar{\theta}$(mean phase deviation) was calculated across both of the other participants in the group to allow for an overall measure of synchrony as experienced by that participant.

It was expected that participants in the synchrony conditions would have little variance in phase differences with the overall distribution centred on 0° (i.e., participants were generally in phase with each other). Participants in the asynchrony conditions were expected to have no stable phase relationship with large variance in phase differences. The sequential conditions were also expected to have little variance in phase differences, but the overall distribution of phase differences would be centred on 240° (i.e. coordinated but consistently out of phase with each other). Note that in the sequential condition, one of the participants is actually 120° out of phase and the other 240° out of phase with the participant of interest. As both the matched participants are included in the calculation of a participant’s , this would result in a bimodal distribution, a lower , and therefore suggesting more dispersal than actually present. To create a unimodal distribution, the θ for the participant that would be 120° out of phase was subtracted from 360°.

Results confirmed these expectations. For the synchrony condition, $\bar{R}$(variance in phase difference) for all participants was above 0.9 suggesting minimal variance in phase angle (varies from 0 to 1 and is the inverse of the circular variance such that an of 1 means that all data points are coincident [1]). The Rayleigh and V-tests for all participants in the synchrony condition were highly significant (*p* < .001) indicating that the data was unimodal and centred on 0°. For the sequential condition for all participants was above 0.8. The Rayleigh and V-tests for all participants in the sequential conditions were highly significant (*p* < .001) indicating that the data was unimodal and centred on 240°. for all participants in the asynchrony groups was considerable smaller (< 0.3) than for synchrony and sequential groups suggesting that participants in the asynchrony groups had minimal entrainment to each other. The Rayleigh and V-tests for all participants in the asynchrony individual goal condition was not significant (*p* > .05) indicating that the data was uniformly distributed.for all participants in the asynchrony individual goal condition was also below 0.05. However, the Rayleigh test for 5 participants in the asynchrony group goal condition was significant (*p* < .001), suggesting the data was not uniform. V-tests examining if the data was centred on a mean of 0° were not significant. V-tests examining if the data was centred on a mean of 180° were significant for all five participants. This suggests that to help stay out of time, some participants moved anti-phase with another participant. However, visual inspection of the distribution of θ for these participants showed that θ was still highly dispersed compared to participants in the synchrony and sequential conditions. This indicates that although there was a slight trend towards anti-phase coordination with these participants, the movement was still highly asynchronous.

Because is on a linear scale from 0 to 1, linear parametric statistics were also used to test for differences in measured synchrony in the overall mean between conditions [2]. The overall mean for the synchrony group goal condition (*M* = 0.94, *SD* = 0.02) was significantly lower than the overall mean for the synchrony individual goal condition (*M* = 0.96, *SD* = 0.03), *t*(26) = 2.95, *p* = .01, *d* = 1.15, suggesting participants were not quite as synchronous in the group goal condition. The overall mean for the sequential group goal condition (*M* = 0.97, *SD* = 0.02) was not significantly different to the overall mean for the sequential individual goal condition (*M* = 0.95, *SD* = 0.06), Welsh’s *t*(11.14) = 1.16, *p* = 0.27, *d* = 0.45, indicating no difference between these two conditions. The overall mean for the asynchrony group goal condition (*M* = 0.12, *SD* = 0.08) was significantly higher than the overall mean for the asynchrony individual goal condition (*M* = 0.03, *SD* = 0.01), Welsh’s *t*(13.95) = 4.31, *p* < .01, *d* = 1.58, suggesting that participants were not quite as good at being asynchronous in the individual goal condition. Comparing the overall mean across movement conditions, both the synchrony condition (*M* = 0.95, *SD* = 0.03) and the sequential condition (*M* = 0.96, *SD* = 0.04) were significantly higher than the asynchrony condition (*M* = 0.07, *SD* = 0.07), Welsh’s *t*(32.16) = 58.28, *p* < .01, *d* = 16.34 and Welsh’s *t*(42.37) =53.57, *p* < .01, *d* = 15.61 for synchrony and sequential respectively. The synchrony condition was not significantly different from the sequential condition (*M* = 0.96, *SD* = 0.04), *t*(51) = 1.40, *p* =.17, *d* = .39.

In summary, although the synchrony group goal condition was not as synchronous as the synchrony individual goal condition and the asynchrony group goal was not as asynchronous as the asynchrony individual goal condition, these differences were minor compared to the differences between movement conditions. Therefore, the movement manipulation was effective and showed the expected behavioural patterns.

**Perceived synchrony**

We also tested the movement manipulation by examining whether participants perceived more synchrony in the synchrony conditions. A 3 (movement) x2 (goal) factorial ANOVA on perceived synchrony revealed the predicted main effect of synchrony on perceived synchrony, *F*(2,76) = 42.86, *p* < .01, *η*_p_^2^ = .53. No main effect of goal was found on perceived synchrony , *F*(1,76) = 0.83, *p* = .37, *η*_p_^2^ = .01. However, there was a significant Movement x Goal interaction on perceived synchrony, *F*(2,76) = 4.59, *p* = .01, *η*_p_^2^ = .11. Exploration of this interaction using Gabriel post-hoc tests found that for the group goal conditions, both sequential (*M* = 4.02, *SD* = 1.11) and asynchrony (*M* = 3.25, *SD* = 1.03) were significantly different from synchrony (*M* = 5.27, *SD* = 0.90; *p* = .01, *d* = 1.24 and *p* < .01, *d* = 2.09 respectively). Furthermore, the sequential condition was not significantly different from the asynchrony condition (*p* = .15, *d* = 0.72). For the individual goal conditions, asynchrony (*M* = 2.33, *SD* = 0.90) was significantly different from synchrony (*M* = 4.98, *SD* = 0.65, *p* < .01, *d* = 3.38). Sequential (*M* = 4.65, *SD* = 1.05) was significantly different from asynchrony (p < .01, d = 2.37), but was not significantly different from synchrony (*p* = .70, *d* = 0.38). Independent t-tests comparing group goal to individual goal for each of the three levels of the movement variable only found a significant difference between group goal and individual goal for the asynchrony condition, *t*(25) = 2.469, *p* = .02, *d* = 0.95, with the asynchrony individual goal condition having a significantly lower perceived synchrony than the asynchrony group goal condition. These data suggest that in terms of perceived synchrony, the manipulation worked for the synchrony and asynchrony conditions. However, the sequential condition still appears to be perceived as reasonably synchronous in the individual goal condition. The lower level of perceived synchrony for the asynchrony individual goal condition compared to the asynchrony group goal condition could be because participants were better at moving asynchronously when they had the beat to guide them.

**Perceived cooperation**

We used perceived cooperation to check the goal manipulation. A priori, we would expect the group goal conditions would be judged as more cooperative than the individual goal conditions, given that participants were explicitly instructed to act in a coordinated fashion. Subsequent analysis supported this prediction. A 3 (movement) x2 (goal) factorial ANOVA on perceived cooperation revealed a main effect of goal on perceived cooperation, *F*(1,76) = 6.02, *p* = .02, *η*_p_^2^ = .07 with the group goal conditions showing a significantly higher mean for perceived cooperation (*M* = 4.39, *SD* = 1.38) than individual goal conditions (*M* = 3.61, *SD* = 1.44; *d* = 0.55). Notably, the analysis also found a main effect of synchrony on perceived cooperation, *F*(2,76) = 7.36, *p* < .01, *η*_p_^2^ = .16. Gabriel post-hoc tests revealed the synchrony conditions produced a significantly higher mean for perceived cooperation (*M* = 4.54, *SD* = 1.22) than the asynchrony conditions (*M* = 3.26, *SD* = 1.61; *p* < .01, *d* = 0.90). The sequential (*M* = 4.22, *SD* = 1.24) condition also yielded a significantly higher mean for perceived cooperation than the asynchrony condition (*p* = .03, *d* = 0.67). No such differences were found when comparing the synchrony and sequential conditions (*p* = .86, *d* = 0.26). Nor did the Movement x Goal interaction yield significant differences, *F*(2,76) = 2.27, *p* = .27, *η*_p_^2^ = .03. This analysis confirms that the use of an explicit goal instruction (shared intentionality) increased perceived cooperation. However, it also suggests that synchronous movement results in greater perceived cooperation than asynchronous movement.

**Perceived difficulty**

Finally we used perceived difficulty of the movements to determine whether moving without a metronome was more difficult than moving with a metronome. A 3 x 2 ANOVA found a significant Movement x Goal interaction on perceived difficulty, *F*(2,76) = 5.14, *p* = .01, *η*_p_^2^ = .12. Independent t-tests comparing group goal to individual goal for each of the three levels of the movement variable only found a significant difference between the asynchrony individual goal condition and the asynchrony group goal condition, *t*(16.53) = 2.64, *p* = .02, *d* = 1.85, with the asynchrony group goal condition having a significantly higher perceived difficulty (*M* = 3.29, *SD* = 0.90) than the asynchrony individual goal condition (*M* = 1.77, *SD* = 0.73). Although the asynchrony individual goal condition was rated as more difficult than the asynchrony group goal condition the mean was still on the ‘easy’ half of the scale. This suggests that participants found it possible to intentionally move out of time without much effort. Neither of the main effects of movement or goal significantly affected perceived difficulty, *F*_max_ = 1.08, *p* = .34.

**References**

1. Fisher (1993) Circular Statistics. Cambridge, UK: Cambridge University Press.

2. Kirschner S, Tomasello M (2009) Joint drumming: Social context facilitates synchronization in preschool children. Journal of Experimental Child Psychology 102: 299–314. doi:10.1016/j.jecp.2008.07.005.
